# Trends in Prevalence of Tobacco Use by Sex and Socioeconomic Status in 22 Sub-Saharan African Countries, 2003-2019

**DOI:** 10.1001/jamanetworkopen.2021.37820

**Published:** 2021-12-08

**Authors:** Chandrashekhar T. Sreeramareddy, Kiran Acharya

**Affiliations:** 1 Department of Community Medicine, International Medical University School of Medicine, Kuala Lumpur, Malaysia; 2 New ERA Kalopul, Rudramati Marg, Kathmandu, Nepal

## Abstract

**Importance:**

Tobacco companies have shifted their marketing and production to sub-Saharan African countries, which are in an early stage of the tobacco epidemic.

**Objective:**

To estimate changes in the prevalence of current tobacco use and socioeconomic inequalities among male and female participants from 22 sub-Saharan African countries from 2003 to 2019.

**Design, Setting, and Participants:**

Secondary data analyses were conducted of sequential Demographic and Health Surveys in 22 sub-Saharan African countries including male and female participants aged 15 to 49 years. The baseline surveys (2003-2011) and the most recent surveys (2011-2019) were pooled.

**Exposures:**

Household wealth index and highest educational level were the markers of inequality.

**Main Outcomes and Measures:**

Sex-specific absolute and relative changes in age-standardized prevalence of current tobacco use in each country and absolute and relative measures of inequality using pooled data.

**Results:**

The survey samples included 428 197 individuals (303 232 female participants [70.8%]; mean [SD] age, 28.6 [9.8] years) in the baseline surveys and 493 032 participants (348 490 female participants [70.7%]; mean [SD] age, 28.5 [9.4] years) in the most recent surveys. Both sexes were educated up to primary (35.7%) or secondary school (40.0%). The prevalence of current tobacco use among male participants ranged from 6.1% (95% CI, 5.2%-6.9%) in Ghana to 38.3% (95% CI, 35.8%-40.8%) in Lesotho in the baseline surveys and from 4.5% (95% CI, 3.7%-5.3%) in Ghana to 46.0% (95% CI, 43.2%-48.9%) in Lesotho during the most recent surveys. The decrease in prevalence ranged from 1.5% (Ghana) to 9.6% (Sierra Leone). The World Health Organization target of a 30% decrease in smoking was achieved among male participants in 8 countries: Rwanda, Nigeria, Ethiopia, Benin, Liberia, Tanzania, Burundi, and Cameroon. For female participants, the number of countries having a prevalence of smoking less than 1% increased from 9 in baseline surveys to 16 in the most recent surveys. The World Health Organization target of a 30% decrease in smoking was achieved among female participants in 15 countries: Cameroon, Namibia, Mozambique, Mali, Liberia, Nigeria, Burundi, Tanzania, Malawi, Kenya, Rwanda, Zimbabwe, Ethiopia, Burkina Faso, and Zambia. For both sexes, the prevalence of tobacco use and the decrease in prevalence of tobacco use were higher among less-educated individuals and individuals with low income. In both groups, the magnitude of inequalities consistently decreased, and its direction remained the same. Absolute inequalities were 3-fold higher among male participants, while relative inequalities were nearly 2-fold higher among female participants.

**Conclusions and Relevance:**

Contrary to a projected increase, tobacco use decreased in most sub-Saharan African countries. Persisting socioeconomic inequalities warrant the stricter implementation of tobacco control measures to reach less-educated individuals and individuals with low income.

## Key Points

**Question:**

How have the prevalence of current tobacco use and socioeconomic inequalities in tobacco use changed in 22 countries of the sub-Saharan Africa region?

**Findings:**

Secondary data analyses of Demographic Health Surveys in 22 sub-Saharan African countries showed that current tobacco use had significantly decreased; the decrease was steeper among female participants and among less-educated individuals and individuals with low income of both sexes. Contrary to projections, current tobacco use had significantly decreased to achieve the World Health Organization target of 30% reduction in one-third of the 22 sub-Saharan countries.

**Meaning:**

This study suggests that stricter tobacco control measures may be helpful, particularly among those at higher risk.

## Teaser

This secondary analysis of data from the Demographic and Health Surveys in 22 sub-Saharan African countries estimates changes in the prevalence of current tobacco use and socioeconomic inequalities among male and female participants from 2003 to 2019.

## Introduction

Worldwide tobacco use has caused more than 7 million deaths since 1990.^1^ Most of the world’s tobacco users live in low- and middle-income countries (LMICs).^2^ Tobacco use is decreasing globally; however, the rates of decrease are unequal by geography, sex, and age.^2,3^ Decreasing tobacco use has been attributed to the scale-up of tobacco control interventions, particularly in high-income countries rather than in LMICs that have a higher tobacco-associated disease burden that continues to increase.^2^ The World Health Organization’s (WHO’s) Framework Convention on Tobacco Control (FCTC), which guides tobacco control measures, has been ratified by most countries.^4^ Therefore, it is important to assess the changes in the prevalence of tobacco use via population-based surveys to assess the effect of tobacco control measures and to inform tobacco control policy making.^2^

As tobacco use continues to decrease in high-income countries, the tobacco industry has increased marketing and production in sub-Saharan Africa (SSA).^5^ As a result, the prevalence of tobacco use in SSA countries was expected to undergo the largest increase^6^ because the SSA region is still in an early stage of the tobacco epidemic.^7^ Projections were made that tobacco use in SSA countries would be higher than in LMICs in other regions by 2025.^8^ For SSA countries, comparable baseline, nationally representative estimates of tobacco use are limited to Global Adult Tobacco Survey estimates in 9 countries,^9^ Demographic and Health Survey (DHS) estimates for 30 of the 46 SSA countries,^10^ and systematic reviews that do not include nationally representative survey data.^11,12,13^ Demographic and Health Surveys are useful data sources for country-level prevalence estimates of current tobacco use^9^ and socioeconomic inequalities in tobacco use.^14^

The WHO Global Action Plan for the Prevention and Control of Noncommunicable Diseases (NCD) targets 5 goals for a 30% relative reduction in current tobacco use among individuals aged 15 years or older by 2025.^15^ Country-level monitoring of inequalities in tobacco use is also critical for a tobacco control policy to reduce inequalities and achieve universal health coverage under Sustainable Development Goals.^16^ Literature on the trends in the inequalities in tobacco use in LMICs^17^ is limited, which underscores the need for monitoring of socioeconomic inequalities in tobacco use and for progress toward the WHO NCD target.^15^ We report sex-specific changes in the age-standardized prevalence of current tobacco use, as well as socioeconomic inequalities in 22 SSA countries for which at least 2 sequential sets of DHS data were available. Using aggregate (country-level) data, we explored the association of the tobacco control policy score with the relative percentage change in the prevalence of tobacco use.

## Methods

### Design and Data Source

We conducted secondary data analyses of sequential DHSs of at least 5-year intervals from 2003 onward for baseline surveys and 2011 onward for the most recent surveys (Table 1). In brief, DHSs are a series of cross-sectional, nationally representative household surveys that collect reliable data on population, health, and nutrition usually every 5 years to monitor change over time. Households are selected by a 2-stage, stratified cluster-sampling technique; the clusters are selected from both urban and rural areas using a probability-proportional-to-size technique followed by a random selection of households from within the selected clusters. Trained interviewers gather data from all eligible male and female residents aged 15 to 49 years according to standard protocols on pretested questionnaires in local languages. For quality-control purposes and to minimize nonresponse, field supervisors ensure that guidelines are strictly adhered to. Male participants are interviewed in a subsample of households selected for the female participants’ survey. Informed verbal consent was sought from all survey participants.^18^ International Medical University exempted this study from ethical approval because all deidentified data are available in the public domain. This survey study follows the American Association for Public Opinion Research (AAPOR) reporting guideline.

**Table 1.** Change in Age-Standardized Prevalence Estimates of Any Tobacco Use Among Male and Female Participants

Country (survey years)	Change in prevalence of any tobacco use among male participants, estimate (95% CI)				Change in prevalence of any tobacco use among female participants, estimate (95% CI)				MPOWER score
	Baseline survey	Most recent survey	Point change, No. (% change)	*P* value^a^	Baseline survey	Most recent survey	Point change, No. (% change)	*P* value^a^	
Burkina Faso (2003-2010)	NA	22.6 (21.1-24.0)	NA	NA	4.6 (4.0-5.2)	3.0 (2.5-3.5)	–1.6 (–35.4)	.006	18
Benin (2012-2018)	9.4 (8.2-10.5)	6.4 (5.5-7.2)	–3.0 (–32.1)	<.001	0.7 (0.5-0.9)	0.8 (0.4-1.1)	0.1 (11.8)	≥.99	19
Burundi (2010-2017)	17.4 (15.9-18.9)	10.7 (9.7-11.6)	–6.8 (–38.8)	<.001	9.4 (8.3-10.4)	2.2 (1.9-2.5)	–7.2 (–76.9)	<.001	12
Democratic Republic of Congo (2007-2014)	NA	24.9 (22.5-27.2)	NA	NA	2.4 (1.5-3.4)	2.7 (2.1-3.3)	0.3 (10.3)	≥.99	21
Cameroon (2011-2018)	14.5 (13.2-15.7)	8.1 (7.1-9.2)	–6.3 (–43.7)	<.001	0.6 (0.4-0.8)	0.0 (0.0-0.1)	–0.6 (–96.7)	<.001	20
Ethiopia (2011-2016)	6.8 (5.9-7.8)	4.7 (3.6-5.0)	–2.2 (–31.9)	.003	0.6 (0.4-0.8)	0.3 (0.1-0.5)	–0.3 (–54.8)	.007	17
Ghana (2008-2014)	6.1 (5.2-6.9)	4.5 (3.7-5.3)	–1.5 (–25.5)	.01	0.3 (0.1-0.4)	0.2 (0.1-0.4)	0.0 (–11.5)	.73	21
Kenya (2009-2014)	18.5 (15.9-21.0)	16.4 (15.3-17.5)	–2.1 (–11.2)	.002	1.3 (0.7-1.8)	0.3 (0.3-0.4)	–1.0 (–74.8)	.001	21
Liberia (2007-2013)	14.2 (12.7-15.7)	9.3 (7.8-10.7)	–4.9 (–34.6)	<.001	2.3 (1.7-2.8)	0.4 (0.3-0.5)	–1.9 (–84.1)	<.001	14
Lesotho (2009-2014)	38.3 (35.8-40.8)	46.0 (43.2-48.9)	7.7 (20.1)	<.001	8.3 (7.5-9.2)	6.3 (5.5-7.2)	–2.0 (–24.1)	.001	15
Mali (2013-2018)	16.0 (14.5-17.4)	11.9 (10.5-13.2)	–4.1 (–25.5)	<.001	0.9 (0.7-1.2)	0.1 (0.1-0.2)	–0.8 (–85.9)	<.001	18
Malawi (2010-2016)	17.2 (15.9-18.5)	12.6 (11.4-13.8)	–4.6 (–26.7)	<.001	0.9 (0.8-1.1)	0.2 (0.2-0.3)	–0.7 (–75.0)	<.001	13
Mozambique (2003-2011)	24.7 (21.9-27.5)	19.7 (17.7-21.6)	–5.0 (–20.4)	.003	6.0 (5.1-6.8)	0.6 (0.5-0.8)	–5.3 (–89.6)	<.001	18
Nigeria (2013-2018)	7.2 (6.5-7.9)	5.0 (4.5-5.4)	–2.2 (–31.2)	<.001	0.3 (0.2-0.4)	0.1 (0.01-0.1)	–0.3 (–83.3)	<.001	17
Niger (2006-2012)	NA	15.7 (13.9-17.5)	NA	NA	1.9 (1.1-2.8)	1.7 (1.1-2.4)	–0.2 (–10.3)	.72	20
Namibia (2007-2013)	23.8 (21.5-26.0)	20.0 (18.0-22.0)	–3.8 (–15.8)	.02	7.1 (6.2-8.1)	0.6 (0.5-0.8)	–6.5 (–91.4)	<.001	20
Rwanda (2008-2015)	13.7 (12.6-14.8)	9.6 (8.7-10.5)	–4.1 (–30.2)	<.001	2.9 (2.6-3.3)	0.9 (0.7-1.1)	NA	<.001	12
Sierra Leone (2013-2019)	26.1 (23.8-28.4)	21.4 (20.1-22.7)	–9.6 (–36.8)	<.001	3.1 (2.5-3.7)	4.6 (4.0-5.2)	0.7 (22.2)	<.001	13
Tanzania (2012-2016)	20.3 (18.5-22.4)	13.3 (11.8-14.8)	–7.0 (–34.6)	<.001	1.4 (1.0-1.7)	0.3 (0.2-0.6)	–0.8 (–69.1)	<.001	NA
Zambia (2014-2018)	18.8 (17.7-19.9)	18.6 (17.4-19.9)	–0.2 (–0.9)	.84	1.3 (1.0-1.6)	0.9 (0.7-1.1)	–0.4 (–29.9)	.03	16
Zimbabwe (2010-2015)	22.5 (21.0-24.0)	17.7 (16.6-18.9)	–4.8 (–21.3)	<.001	0.4 (0.3-0.6)	0.1 (0.1-0.2)	–0.3 (–67.4)	<.001	15
Senegal (2005-2011)	18.2 (16.4-19.9)	13.5 (12.4-14.6)	–5.7 (–31.2)	<.001	0.2 (0.1-0.3)	0.9 (0.6-1.1)	0.7 (377.8)	.002	25

Abbreviations: MPOWER, (1) monitor tobacco consumption and the effectiveness of preventive measures; (2) protect people from tobacco smoke; (3) offer help to quit tobacco use; (4) warn about the dangers of tobacco; (5) enforce bans on tobacco advertising, promotion, and sponsorship; and (6) raise taxes on tobacco; NA, not available.

^a^ Wald statistics were used to estimate the statistical significance of difference in the point estimates between the 2 surveys.

### Outcome Variables

The definition of “current tobacco use” was constructed based on 3 survey questions that were the same in all surveys, except that the response options for tobacco products were different in some countries based on the prevailing tobacco products consumed in that country: (1) Do you currently smoke cigarettes? (Yes or no.) (2) Do you currently smoke or use any other type of tobacco? (Yes or no.) (3) What (other) type of tobacco do you currently smoke or use? (Pipe, chewing tobacco, snuff, etc.)

Current tobacco use (eg, cigarettes, pipe, cigars, chewing, or snuff) was assumed if the response was “yes” for questions 1 and/or 2 and if the respondents indicated the use of any listed tobacco products for question 3. Dual tobacco product users were included under current tobacco user, and we did not report separately on the prevalence of smoking and smokeless tobacco use.

### Markers and Measures of Inequality

The household wealth index and the highest level of education completed were used as markers of inequality. The wealth index is a score generated by principal component analysis using socioeconomic factors and the presence of items such as a television or radio, which classifies households into 5 quintiles (the first quintile is the 20% of households with the lowest income, and the fifth quintile is the 20% of households with the highest income),^19^ allowing for cross-country comparisons and time-trend analyses across socioeconomic groups.^20^ Information on number of years of schooling was used to create classifications of no education (0 years), primary education (1-5 years), secondary education (6-10 years), or higher education (>10 years; university or vocational education after school). To avoid misinterpretations of measures of health inequality caused by different population sizes, choice of reference category, and the scale of measurement,^21^ we estimated both absolute and relative measures of inequality (namely, the slope index of inequality [SII] and the relative index of inequality [RII]).^22^

### MPOWER Score

*MPOWER* stands for 6 effective strategies for fighting the global tobacco epidemic: (1) monitor tobacco consumption and the effectiveness of preventive measures; (2) protect people from tobacco smoke; (3) offer help to quit tobacco use; (4) warn about the dangers of tobacco; (5) enforce bans on tobacco advertising, promotion, and sponsorship; and (6) raise taxes on tobacco. MPOWER data were extracted from the WHO global tobacco epidemic reports for the year closest to the most recent survey. For each of the 6 measures, a score of 1 is ascertained if data were lacking or no recent data (since 2009) were available or data were not both recent and representative (national population), whereas scores of 2 to 4 (for monitor [M]) and scores of 2 to 5 (for protect [P], offer [O], warn [W], enforce [E], and raise taxes [R]) represent a scale from weakest to strongest level of tobacco control policy in that country.^23^ The highest possible MPOWER score after summing the scores for each dimension is 29.

### Statistical Analysis

For each country and survey year, we calculated sex-specific, age-standardized prevalence estimates and their 95% CIs (applying the WHO standard population^24^) of current tobacco use. Sample weights were considered for the complex sampling design of the DHS. The absolute difference in prevalence rates and the percentage change in prevalence during the most recent survey from the baseline survey was calculated. To test the statistical significance of the change in the estimate between the surveys, we calculated Wald statistics (difference/estimated SE) using aggregate data for each survey (ie, prevalence estimates and 95% CIs for the baseline and most recent surveys). Sex-specific data available for male participants (19 countries) and female participants (22 countries) were pooled separately for the baseline and most recent surveys. For pooled data for baseline surveys and most recent surveys, we estimated the measures of inequality and the prevalence of current tobacco use by educational attainment and wealth categories. We calculated the change in prevalence estimates and the measures of inequality between the baseline and most recent surveys in each population subgroup.

For the measures of inequality, on each pooled data set, we first calculated ridit scores indicating the cumulative proportion of the population at each socioeconomic level, ordered from lowest to highest.^25^ Individuals with the same score were assigned a mean rank. By regression analyses using current tobacco use as an outcome variable and the ridit score as the exposure variable, we estimated the difference in log odds of current tobacco use for a 1-unit change in socioeconomic rank (ie, from the bottom [0] to the top [1] of the socioeconomic scale). We used our model coefficients as marginal estimates with SEs of current tobacco use at the bottom and top of the socioeconomic distribution, and we used linear and nonlinear contrasts to calculate SII and RII, respectively. The SII is estimated as the expected difference in current tobacco use between the bottom and the top of the socioeconomic distribution, and the RII is the ratio of the same 2 estimates. Thus, if current tobacco use decreases with increasing socioeconomic position, then the SII is greater than 0 and the RII is greater than 1, whereas if current tobacco use increases with increasing socioeconomic position, then the SII is less than 0 and the RII is less than 1. All *P* values were from 2-sided tests and results were deemed statistically significant at *P* < .05.

## Results

Data were available for both sexes in 19 of the 22 SSA countries. For Burkina Faso, the Democratic Republic of Congo, and Niger, either male participants were not interviewed or tobacco use questions were not asked in the survey of male participants. The survey samples included 428 197 individuals (303 232 female participants [70.8%]; mean [SD] age, 28.6 [9.8] years) in the baseline surveys and 493 032 participants (348 490 female participants [70.7%]; mean [SD] age, 28.5 [9.4] years) in the most recent surveys (eTable in the Supplement). The distribution of male participants by wealth and education was comparable between the 2 surveys. Nearly 16.0% of male participants belonged to the households with the lowest income and 25.0% to the households with the highest income. The distribution of female participants by wealth was comparable to that of male participants in both surveys. Both sexes were educated up to primary school (35.7%) or secondary school (40.0%). In both surveys, approximately 74.0% of male participants were educated up to secondary school. Approximately 65.0% of female participants were not educated or were educated up to primary school only.

Table 1 shows age-standardized prevalence percentage estimates and the change in prevalence rates. The intervals ranged from 4 to 8 years. The annual rate of change was higher among female participants (ranging from 2.1% in Niger to 13.8% in Cameroon) than male participants (ranging from 0.2% in Zambia to 6.4% in Ethiopia). Among male participants, tobacco use rates varied from 6.1% (95% CI, 5.2%-6.9%) in Ghana to 38.3% (95% CI, 35.8%-40.8%) in Lesotho in the baseline surveys and from 4.5% (95% CI, 3.7%-5.3%) in Ghana to 46.0% (95% CI, 43.2%-48.9%) in Lesotho during the most recent surveys. Except for Lesotho, which also had the highest rate of tobacco use, in all other SSA countries except Zambia, tobacco use rates had significantly decreased in terms of prevalence rates ranging from 1.5% (Ghana) to 9.6% (Sierra Leone). The highest percentage decrease of 43.7% was in Cameroon (from 14.5% to 8.1%) between 2011 and 2018. Among male participants, the lowest percentage decrease occurred in Kenya (11.2%; 2009-2014). For male participants, Lesotho was an exception, having the highest prevalence and percentage increase of 7.7% (38.8% to 46.0%) between 2009 and 2014.

Among female participants, tobacco use rates were less than 1% in 9 countries during baseline surveys (2003-2012), which increased to 16 during the most recent surveys (2011-2019) (Table 1). In 4 countries—Burundi (9.4%; 95% CI, 8.3%-10.4%), Lesotho (8.3%; 95% CI, 7.5%-9.2%), Namibia (7.1%; 95% CI, 6.2%-8.1%), and Mozambique (6.0%; 95% CI, 5.1%-6.8%)—the prevalence of tobacco use was greater than 5% at the baseline survey (2006-2013). Benin, the Democratic Republic of Congo, Ghana, and Niger experienced nearly a 10% decrease between the baseline and most recent surveys, whereas in Cameroon, Namibia, and Mozambique, tobacco use decreased by nearly 90%, and the prevalence was less than 1.0% during the most recent surveys. The only exception was an increase from 0.2% at baseline to 0.7% in the most recent survey among female participants in Senegal.

Among female participants, 15 of the 22 countries exceeded the WHO NCD target by a large margin, while Zimbabwe achieved a 30% reduction in 2018. However, for male participants, the target was reached in only 8 countries (Rwanda, Nigeria, Ethiopia, Benin, Liberia, Tanzania, Burundi, and Cameroon). Mali, Malawi, Sierra Leone, and Ghana experienced nearly a 25% decrease in tobacco use among men (Figure). The MPOWER scores in most of the 22 SSA countries ranged from 12 to 25; in 7 countries, MPOWER scores were less than 15. The Spearman correlation showed a moderate coefficient for both male (0.25) and female (0.33) participants, which were not significant.

**Figure.**
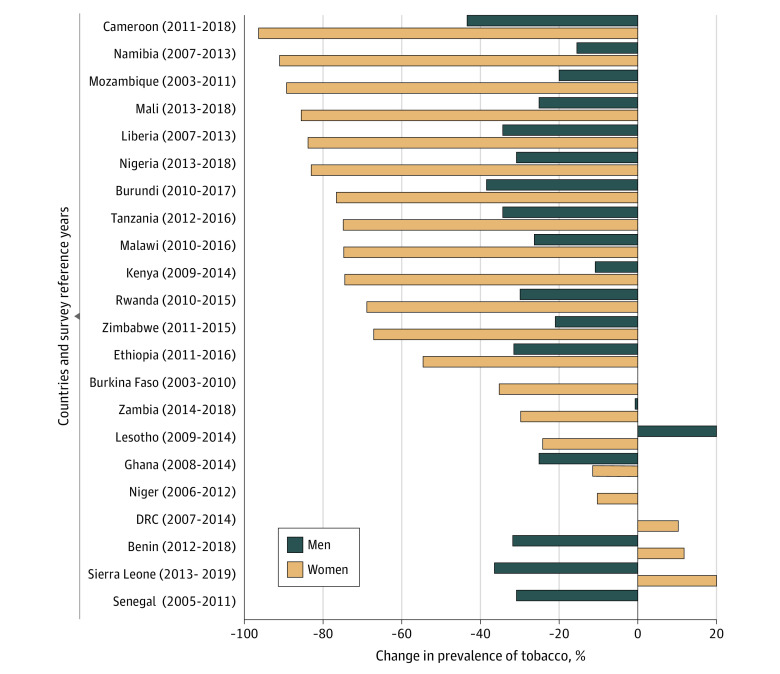
Achievement of World Health Organization Target of 30% Relative Reduction in Prevalence of Current Tobacco Use Female participants from Senegal were excluded from the bar chart because the prevalence of tobacco use increased by 378% and was considered an outlier. DRC indicates Democratic Republic of Congo.

Table 2 shows sex-specific changes in the prevalence of tobacco use (pooled data) disaggregated by educational attainment, wealth, and age groups. Tobacco use by 3 age groups (15-24, 25-34, and 35-49 years) progressively increased. Overall, the percentage decrease among male participants was only 6.8% (ie, from 14.1 [95% CI, 13.8-14.5] to 13.2 [95% CI, 12.8-13.6] percentage points). However, the percentage decrease among female participants was 23.5% (ie, a decrease from 2.1 [95% CI, 2.0-2.2] to 1.7 [95% CI, 1.6-1.8] percentage points). The magnitude and direction of change varied across the educational groups between male participants and female participants. The decrease was steeper among male participants with no education (−25.4%) and among female participants (−19.2% with no education and −33.3% with primary education). Tobacco use consistently decreased in all wealth groups in both sexes (male participants: −5.4% highest income to –12.2% lowest income; female participants: −22.2% highest income to −15.6% lowest income). However, the percentage decrease among male participants was much higher among the lowest income (−12.2%) and lower income (−12.7%) households by wealth index, whereas among female participants, the percentage decrease was much higher among the lower income (−30.0%) and middle incomes (−31.3%) households by wealth index.

**Table 2.** Prevalence of Tobacco Use and Percentage Change by Education, Wealth Index, and Age Groups

Characteristic	Male participants		% Change	Female participants		% Change
	Baseline, % (95% CI)	Most recent survey, % (95% CI)		Baseline, % (95% CI)	Most recent survey, % (95% CI)	
Overall	14.1 (13.8-14.5)	13.2 (12.8-13.6)	–6.8	2.1 (2.0-2.2)	1.7 (1.6-1.8)	–23.5
Educational level						
No education	16.8 (16.3-17.3)	13.4 (12.6-14.1)	–25.4	3.1 (2.9-3.3)	2.6 (2.4-2.8)	–19.2
Primary	15.2 (14.5-15.9)	16.7 (16.2-17.3)	9.0	2.0 (1.8-2.1)	1.5 (1.4-1.7)	–33.3
Secondary	13.6 (13.1-14.1)	11.4 (10.9-11.9)	–19.3	1.2 (1.1-1.3)	1.2 (1.1-1.3)	0
Higher education	5.5 (5.0-6.0)	5.7 (5.1-6.2)	3.5	0.7 (0.5-0.9)	0.6 (0.4-0.7)	–16.7
Wealth						
Lowest income	20.3 (19.6-21.0)	18.1 (17.2-19.0)	–12.2	3.7 (3.4-4.0)	3.2 (3.0-3.4)	–15.6
Lower income	16.9 (16.3-17.6)	15 (14.3-15.7)	–12.7	2.6 (2.4-2.8)	2.0 (1.9-2.2)	–30.0
Middle income	14.4 (13.8-15.0)	14.2 (13.5-14.9)	–1.4	2.1 (2.0-2.3)	1.6 (1.5-1.7)	–31.3
Higher income	12.3 (11.7-12.8)	11.9 (11.2-12.5)	–3.4	1.6 (1.5-1.7)	1.3 (1.2-1.4)	–23.1
Highest income	9.7 (9.1-10.2)	9.2 (8.7-9.7)	–5.4	1.1 (1.0-1.2)	0.9 (0.8-0.9)	–22.2
Age groups, y						
15-24	6.5 (6.2-6.8)	5.9 (5.6-6.2)	–10.2	0.6 (0.6-0.7)	0.7 (0.6-0.8)	14.3
25-34	18.9 (18.4-19.5)	17.4 (16.8-18.0)	–8.6	2.1 (2.0-2.3)	1.8 (1.7-1.9)	–16.7
35-49	21.9 (21.3-22.5)	21.1 (20.5-21.7)	–3.8	5.4 (5.1-5.7)	3.9 (3.7-4.1)	–38.5

### Educational Inequalities

Table 3 displays the SII and the RII by educational and wealth groups. Tobacco use was higher among the least educated male participants and female participants than those with a higher level of education. Rate differences and absolute inequalities (SII) by educational group were much higher among male participants. During the most recent surveys, in absolute terms, the tobacco use rate increased by approximately 8.3% (95% CI, 7.2%-9.3%) among male participants and 2.4% (95% CI, 2.1%-2.7%) among female participants from the highest education level to the lowest education level. However, during the most recent surveys, the magnitude of the relative inequalities was much higher among female participants despite the much lower tobacco use rates. In relative terms, the tobacco use rate differed by only a factor of 1.9% (95% CI, 1.7%-2.0%) among male participants and by a factor of 4.1% (95% CI, 3.4%-4.8%) among female participants from the highest education level to the lowest (no) education group.

**Table 3.** Socioeconomic Inequalities of Tobacco Use Among Male and Female Participants By Education and Wealth Index

Characteristic	Male participants		Female participants	
	Baseline survey, % (95% CI)	Most recent survey, % (95% CI)	Baseline survey, % (95% CI)	Most recent survey, % (95% CI)
Rate difference				
No education	9.7 (8.9-10.5)	7.7 (6.8-8.6)	2.4 (2.1-2.7)	2.0 (1.8-2.3)
Primary	11.3 (10.6-11.9)	11.1 (10.3-11.8)	1.2 (1.0-1.5)	1.0 (0.8-1.2)
Secondary	8.1 (7.5-8.7)	5.7 (5.0-6.4)	0.5 (0.3-0.7)	0.6 (0.5-0.8)
Higher education	1 [Reference]	1 [Reference]	1 [Reference]	1 [Reference]
SII (95% CI)	8.7 (7.8-9.7)	8.3 (7.2-9.3)	3.0 (2.6-3.4)	2.4 (2.1-2.7)
Rate difference				
Lowest income	10.6 (9.8-11.5)	8.9 (8.0-9.9)	2.6 (2.3-2.9)	2.3 (2.1-2.6)
Lower income	7.3 (6.4-8.1)	5.8 (5.0-6.7)	1.5 (1.2-1.7)	1.2 (1.0-1.4)
Middle income	4.7 (4.0-5.5)	5 (4.2-5.8)	1.1 (0.9-1.3)	0.8 (0.6-0.9)
Higher income	2.6 (1.9-3.4)	2.7 (2.0-3.5)	0.5 (0.3-0.7)	0.4 (0.3-0.6)
Highest income	1 [Reference]	1 [Reference]	1 [Reference]	1 [Reference]
SII (95% CI)	12.8 (11.8-13.8)	10.6 (9.5-11.8)	3.1 (2.7-3.5)	2.7 (2.4-3.0)
Prevalence ratio				
No education	2.8 (2.5-3.0)	2.4 (2.1-2.6)	4.3 (3.2-5.8)	4.7 (3.6-6.1)
Primary	3.0 (2.8-3.3)	3.0 (2.7-3.3)	2.7 (2.0-3.7)	2.8 (2.1-3.6)
Secondary	2.5 (2.3-2.7)	2.0 (1.8-2.2)	1.7 (1.3-2.2)	2.1 (1.6-2.8)
Higher education	1 [Reference]	1 [Reference]	1 [Reference]	1 [Reference]
RII (95% CI)	1.9 (1.7-2.0)	1.9 (1.7-2.0)	4.2 (3.6-5.0)	4.1 (3.4-4.8)
Prevalence ratio				
Lowest income	10.6 (9.8-11.5)	8.9 (8.0-9.9)	3.4 (2.9-3.9)	3.7 (3.3-4.3)
Lower income	7.3 (6.4-8.1)	5.8 (5.0-6.7)	2.4 (2.0-2.7)	2.4 (2.1-2.7)
Middle income	4.7 (4.0-5.5)	5.0 (4.2-5.8)	2.0 (1.7-2.3)	1.9 (1.6-2.2)
Higher income	2.6 (1.9-3.4)	2.7 (2.0-3.5)	1.5 (1.3-1.7)	1.5 (1.3-1.7)
Highest income	1 [Reference]	1 [Reference]	1 [Reference]	1 [Reference]
RII (95% CI)	2.5 (2.3-2.7)	2.3 (2.1-2.5)	4.4 (3.7-5.1)	4.9 (4.3-5.7)

Abbreviations: RII, relative index of inequality; SII, slope index of inequality.

From the baseline surveys to the most recent surveys, educational absolute inequalities (SII) in tobacco use had decreased marginally among both male participants (from 8.7% [95% CI, 7.8%-9.7%] to 8.3% [95% CI, 7.2%-9.3%]) and female participants (from 3.0% [95% CI, 2.6%-3.4%] to 2.4% [95% CI, 2.1%-2.7%]) (Table 3). On the other hand, educational relative inequalities (RII) in tobacco use did not change from baseline to the most recent surveys among male participants (1.9% [95% CI, 1.7%-2.0%]) and was nearly the same among female participants (from 4.2% [95% CI, 3.6%-5.0%] to 4.1% [95% CI, 3.4%-4.8%]). The magnitude of relative inequalities among female participants was 2-fold higher than male participants (4.2% vs 1.9%), while its direction remained the same in both sexes.

### Wealth-Related Inequalities

Wealth-related tobacco use inequalities were greater among those in the lowest income quintile and were of a smaller magnitude for both sexes. However, among male participants, the magnitude of wealth-related tobacco use inequalities (both SII and RII) was slightly higher than educational inequalities. Among female participants, the magnitude of wealth-related tobacco use inequalities (both SII and RII) was nearly the same as educational inequalities. Between surveys, both the SII (from 12.8% [95% CI, 11.8%-13.8%] to 10.6% [95% CI, 9.5%-11.8%]) and the RII (from 2.5% [95% CI, 2.3%-2.7%] to 2.3% [95% CI, 2.1%-2.5%]) slightly decreased among male participants, whereas for female participants, the SII (from 3.1% [95% CI, 2.7%-3.5%] to 2.7% [95% CI, 2.4%-3.0%]) marginally decreased, and the RII (from 4.4% [95% CI, 3.7%-5.1%] to 4.9% [95% CI, 4.3%-5.7%]) increased. Like educational inequalities, the wealth-related RII was also nearly 2-fold higher among female participants than male participants (4.9% vs 2.3%).

## Discussion

Our results confirm that the prevalence of current tobacco use was low in most of the SSA countries and that the sex differentials were wide. Tobacco use has decreased for both sexes, but at a steeper rate among female participants, less educated subgroups, and lower income subgroups. The WHO NCD target was reached in 16 countries for female participants and 9 countries for male participants, and many other countries are on track to reach this goal. In both surveys, the SII and the RII were higher among those in the highest income quintile and higher among those with a higher educational level and decreased marginally. The magnitude of inequalities was moderate and consistent in direction. The magnitude of absolute inequalities was 3-fold higher among male participants, while relative inequalities were 2-fold higher among female participants.

Country-level estimates are comparable to those reported in systematic analyses of multisource global data,^8,26^ confirming that SSA countries are still in an early stage of the tobacco epidemic. Our estimates are comparable to Global Adult Tobacco Survey data available for 9 SSA countries,^9^ but DHS questions do not distinguish between daily vs nondaily and current vs past tobacco use. However, our estimates include smokeless tobacco, similar to previous reports in SSA countries.^10,27^ Our estimates are not comparable with the smoking prevalence rates reported in systematic reviews that do not include data from national surveys or data on smokeless tobacco use but include nonstandard definitions for smokers and nonstandard survey designs.^11,12,13^

A decreasing trend in 22 SSA countries is consistent with the reports by the Global Burden of Disease Study^3,26^ and the WHO.^8^ However, our results are contradictory to the projected increase in tobacco use among African men in previous studies^6,8^ associated with the increased production and more aggressive marketing in SSA countries.^5^ The increased production and 44% increase in cigarette sales between 1990 and 2012 in at least 22 SSA countries are likely to be offset by the increased number of smokers in the growing population in SSA countries and/or an increase in the number of cigarettes smoked per day. Cigarette production facilities located in Egypt, South Africa, Nigeria, Kenya, Ethiopia, and Algeria have resulted in increased exports as well.^5^ However, to verify whether increased cigarette production and sales resulted in increased prevalence rates of tobacco use in SSA countries, recent comparable country-level data are needed from most countries. The tobacco industry is known to circumvent tobacco control strategies by weakening the laws using various tactics.^28^ However, there is very little information about tobacco industry interference in SSA countries.^29^ Regardless, continued efforts are needed to address tobacco industry interference in SSA countries.^30^

Health inequalities are known to be rooted in social factors and have lead to a societal debate to improve cross-sector policy and the health of vulnerable populations.^31,32^ Socioeconomic patterning in tobacco use is well known.^33,34^ Higher rates of tobacco use among socioeconomically disadvantaged groups has been associated with health and mortality inequalities.^35^ Measuring the magnitude of inequalities is critical to assessing whether the outcome of tobacco control interventions has occurred consistently and equitably across socioeconomic subgroups and not just achieved the overall WHO NCD target.^16^ Even at a lower prevalence and a decreasing trend, sex-specific tobacco use inequalities of low magnitude still exist in SSA countries, as in earlier studies.^10,14^ Recommended monitoring of the progress toward equity goals and targets in the SSA region is limited owing to the unavailability of data for all countries.^16^

Growing populations and emerging economies in SSA have prompted tobacco companies to shift their focus on marketing and production to SSA to increase the demand for their products. However, the decreasing trend is associated with the WHO FCTC, which has redefined tobacco control policies and interventions since 2005.^4^ Despite varying levels of tobacco control measures in SSA countries,^36,37^ decreasing rates of tobacco use are suggestive of the effect of those measures. Regardless of the low MPOWER score in 22 SSA countries, most of those countries are now signatories of the WHO FCTC,^38^ which has established focal points and tobacco control programs at the national level. However, the adoption of FCTC regulations was not uniform across the SSA countries.^39^ The association between the change in the prevalence of tobacco use and the MPOWER scores in just 22 countries was comparable to previous studies^23,40^ but lacked statistical power. Moreover, the MPOWER scores based on the WHO document review indicate only the existence of tobacco control regulations, not their implementation. Nevertheless, our results support the global projections of the decreasing tobacco epidemic, if comprehensive tobacco control policies are fully implemented.^41^

The suboptimal adoption of FCTC regulations^39^and weaker MPOWER scores in SSA countries call for full-scale and stricter implementation of tobacco control regulations to consolidate the decreasing trends.^42^ However, the SSA region may still remain the focus for tobacco industries to expand their consumer markets.^5^ National governments need to address multinational tobacco companies’ interference in undermining tobacco control measures.^43^ More important, country-level data for all of SSA are needed for monitoring the progress toward the WHO NCD target.^15^

### Strengths and Limitations

This study has some strengths, including comparable, nationally representative, sex-specific, age-standardized estimates available at 5-year intervals.^44^ Our study also included sex-specific patterns of change across age, educational, and wealth groups because data on socioeconomic inequalities are critical for tobacco control policy and for monitoring the progress of health outcomes in the population subgroups.^21^

Our study also includes some limitations, including the DHS design and data availability. First, self-reported current tobacco use is likely to be underestimated because participants were not asked about nondaily tobacco use, and not all types of tobacco products were listed as responses. Second, underreporting and social desirability bias in survey research are very likely owing to the stigma surrounding tobacco use in LMICs, particularly among women,^45^ and the DHS did not validate tobacco use by biomarker estimation.^46^ Third, our inequality measures did not adjust for other covariates to evaluate how they were associated with measures of inequality.^14^ Fourth, male participants surveyed among households selected for the survey of female participants lowered the precision for the prevalence estimates and measures of inequalitiy for male participants. Fifth, the lack of more recent data and the use of data from only 22 countries do not represent the current situation in the whole SSA region.

## Conclusions

The findings from the secondary data analyses of DHSs in 22 SSA countries suggest that tobacco use has decreased in approximately one-third of SSA countries and that the WHO NCD have achieved or are close to acheving the target of a 30% reduction in tobacco use. The persistence of socioeconomic inequalities, although decreasing, warrants stronger implementation of tobacco control interventions to reach vulnerable populations. Finally, regular country-level survey data are needed to monitor equitable progress of decreases in tobacco use.

## Notes

Supplement. **eTable.** Sample of Men and Women Surveyed in Two Sequential Surveys in the Countries Included
