# Supplementary material for: Trends in Prevalence of Tobacco Use by Sex and Socioeconomic Status in 22 Sub-Saharan African Countries, 2003-2019
Source: JAMA Netw Open. 2021 Dec 8;4(12):e2137820. doi: 10.1001/jamanetworkopen.2021.37820 (PMC8655603; doi:10.1001/jamanetworkopen.2021.37820)
Supplement: Supplement. — eTable. Sample of Men and Women Surveyed in Two Sequential Surveys in the Countries Included [file jamanetwopen-e2137820-s001.pdf]

Supplementary Online Content

Sreeramareddy CT, Acharya K. Trends in prevalence of tobacco use by sex and socioeconomic status in 22 sub-Saharan African countries, 2003-2019. *JAMA Netw Open.* 2021;4(12):e2137820. doi:10.1001/jamanetworkopen.2021.37820

eTable. Sample of Men and Women Surveyed in Two Sequential Surveys in the Countries Included

This supplementary material has been provided by the authors to give readers additional information about their work.

© 2021 Sreeramareddy CT et al. *JAMA Network Open.*

eTable. Sample of Men and Women Surveyed in Two Sequential Surveys in the Countries Included

**Country (survey years baseline and most recent)**	**Baseline**		**Most recent survey**	
	**Women**	**Men**	**Women**	**Men**
Burkina Faso (2003, 2010)	12,477	3,208.82	17,087	6,499.78
Benin (2012, 2018)	16,599	4,433.42	15,928	6,727
Burundi (2010, 2017)	9,389	3,759.53	17,269	6,687
Congo DRC (2007, 2014)	9,995	4,316.21	18,827	7,755
Cameroon (2011, 2018)	15,426	6,454.56	13,616	6,126
Ethiopia (2011, 2016)	16,515	12,834.33	15,683	11,606
Ghana (2008, 2014)	4,916	4,057.56	9,396	3,869
Kenya (2009, 2014)	8,444	3,257.65	31,079	12,063
Liberia (2007, 2013)	7,092	6,008.97	9,239	4,118
Lesotho (2009, 2014)	7,624	3,007.55	6,621	2,660
Mali (2013, 2018)	10,424	3,795.82	10,519	4,030
Malawi (2010, 2016)	23,020	6,818.37	24,562	7,128
Mozambique (2003, 2011)	12,418	2,489.80	13,745	3,512
Nigeria (2013, 2018)	38,948	17,359	41,821	11,868
Niger (2006, 2012)	9,223	3,100.70	11,160	3,389
Namibia (2007, 2013)	9,804	3,915.00	9,176	4,021
Rwanda (2008, 2015)	13,671	5,687.49	13,497	5,577
Sierra Leone (2013, 2019)	16658	7262	15574	7197
Tanzania (2012, 2016)	19,319	2,527	13,266	3,514
Zambia (2014, 2018)	16,411	13,561.47	13,683	11,177
Zimbabwe (2010, 2015)	9,171	7,109.51	9,955	8,041
Senegal (2005, 2011)	15688	4929	16787	6977
Total	303,232	124,965	348,490	144,542

© 2021 Sreeramareddy CT et al. *JAMA Network Open.*
